# Phosphorylation of AATYK1 by Cdk5 Suppresses Its Tyrosine Phosphorylation

**DOI:** 10.1371/journal.pone.0010260

**Published:** 2010-04-20

**Authors:** Koji Tsutsumi, Tetsuya Takano, Ryo Endo, Mitsunori Fukuda, Toshio Ohshima, Mineko Tomomura, Shin-ichi Hisanaga

**Affiliations:** 1 Department of Biological Sciences, Tokyo Metropolitan University, Hachioji, Tokyo, Japan; 2 Department of Developmental Biology and Neurosciences, Tohoku University, Miyagi, Japan; 3 Department of Life Science and Medical Biological Science, Waseda University, Tokyo, Japan; 4 Meikai Pharmaco-Medical Laboratory (MPL), Meikai University School of Dentistry, Sakado, Saitama, Japan; Universidade Federal do Rio de Janeiro (UFRJ), Brazil

## Abstract

Apoptosis-associated tyrosine kinase 1 (AATYK1), a novel serine/threonine kinase that is highly expressed in the brain, is involved in neurite extension and apoptosis of cerebellar granule neurons; however, its precise function remains unknown. In this study, we investigated the interaction of AATYK1A with Cyclin-dependent kinase 5 (Cdk5)/p35, a proline-directed protein kinase that is predominantly expressed in neurons. AATYK1A bound to the p35 activation subunit of Cdk5 in cultured cells and in mouse brains and colocalized with p35 on endosomes in COS-7 cells. AATYK1A was phosphorylated at Ser34 by Cdk5/p35 *in vitro*, in cultured neurons and in mouse brain. In PC12D cells, Ser34 phosphorylation increased after treatment with nerve growth factor and phosphorylated AATYK1A accumulated in growth cones of PC12D cells. Ser34 phosphorylation suppressed the tyrosine phosphorylation of AATYK1A by Src family kinases. These results suggest a possibility that AATYK1A plays a role in early to recycling endosomes and its function is regulated by phosphorylation with Cdk5 or Src-family kinases.

## Introduction

Apoptosis-associated tyrosine kinases (AATYKs) are a family of protein kinases comprising AATYK1–3 [1]. AATYK1, which was originally isolated, was termed after its increased expression in myeloid precursor cells undergoing apoptosis and its sequence homology to receptor-type tyrosine kinase [2]. However, all AATYK-family kinases are now considered as Ser/Thr kinases that are expressed abundantly in the brain [3], [4], [5], [6]. AATYK1 expresses two splice variants (AATYK1A and AATYK1B) that differ on the presence of the amino-terminal transmembrane sequence of AATYK1B, which is located upstream of the amino-terminal of AATYK1A [1]. AATYK1A comprises 1,317 amino acids and includes an amino-terminal kinase domain and a long carboxy-terminal tail domain. AATYK1 is also known as lemur tyrosine kinase 1 (LMTK1), based on its long carboxy-terminal tail region [7]. Although AATYK1 is involved in neurite extension and low K^+^-induced apoptosis in cerebellar granule neurons [8], [9], its precise neuronal function remains elusive.

Cyclin-dependent kinase 5 (Cdk5) is a proline-directed serine/threonine (Ser/Thr) kinase that is activated by activation subunits p35 or p39, which are predominantly expressed in neurons [10], [11]. Cdk5/p35 plays a role in a variety of neuronal activities, including neuronal migration, neurite extension, endocytic pathway, synaptic plasticity, and neuronal death in neurodegenerative diseases [12], [13], [14]. We reported the binding of the short isoform of AATYK1 to p35 and its phosphorylation by Cdk5 *in vitro* and in cultured cells [15]; however, the interaction of AATYK1A, the major isoform in neurons, with Cdk5/p35 has not been examined. Furthermore, the phosphorylation site and the role of phosphorylation have not been addressed. In addition, we reported recently that AATYK1A associates with recycling endosomes via palmitoylation at the amino-terminal region [16]. This cellular localization is different from that of Cdk5/p35, which reportedly localizes to the Golgi apparatus and plasma membrane [17], [18]. Thus, the interaction of AATYK1A with Cdk5/p35 warrants more detailed examination. Here, we investigated the interaction, binding, and colocalization of AATYK1A with Cdk5/p35 in HEK293 cells, COS-7 cells, PC12D cells, rat brain cortical neurons and mouse brain. We also assessed the Cdk5/p35 phosphorylation site on AATYK1A, as well as its function.

## Results

### Association of AATYK1A with p35 on endosomes in cultured cells

**Figure 1:**
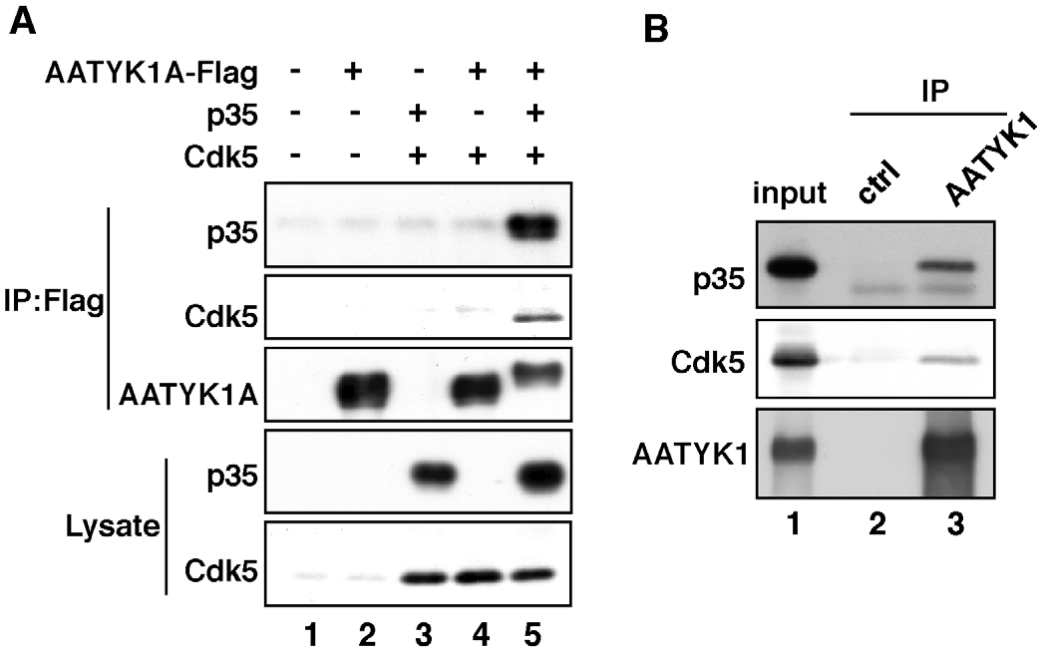
Binding of AATYK1A to Cdk5/p35. (A) Coimmunoprecipitation of Cdk5/p35 with AATYK1A in HEK293 cells. AATYK1A-Flag was coexpressed with p35 and/or Cdk5 in HEK293 cells and immunoprecipitated from cell lysates using the anti-Flag antibody. p35 and Cdk5 were detected in the anti-Flag immunoprecipitates by immunoblotting using anti-p35 and anti-Cdk5 antibodies. (B) Coimmunoprecipitation of Cdk5/p35 from mouse brain extracts using the anti-AATYK1 antibody. AATYK1 was immunoprecipitated from a mouse brain extract (10 weeks). p35 and Cdk5 were detected in the AATYK1 immunoprecipitates using the anti-p35 and anti-Cdk5 antibodies.

AATYK1A tagged with Flag was coexpressed with Cdk5 and/or p35 in HEK293 cells and immunoprecipitated with an anti-Flag antibody from extracts of these cells. Both p35 and Cdk5 were detected in the immunoprecipitates when Cdk5 and p35 were coexpressed (Fig. 1A, lane 5); however, Cdk5 was not found in the immunoprecipitates in the absence of p35 (Fig. 1A, lane 4). Immunoprecipitation of p35 in the absence of Cdk5 has been shown previously (15). All these results indicate that AATYK1A binds to p35 but not to Cdk5. *In vivo* association is shown in Figure 1B. Both p35 and Cdk5 were detected in the immunoprecipitates obtained from brain extracts using the anti-AATYK1 antibody (Fig. 1B, lane 3).

**Figure 2:**
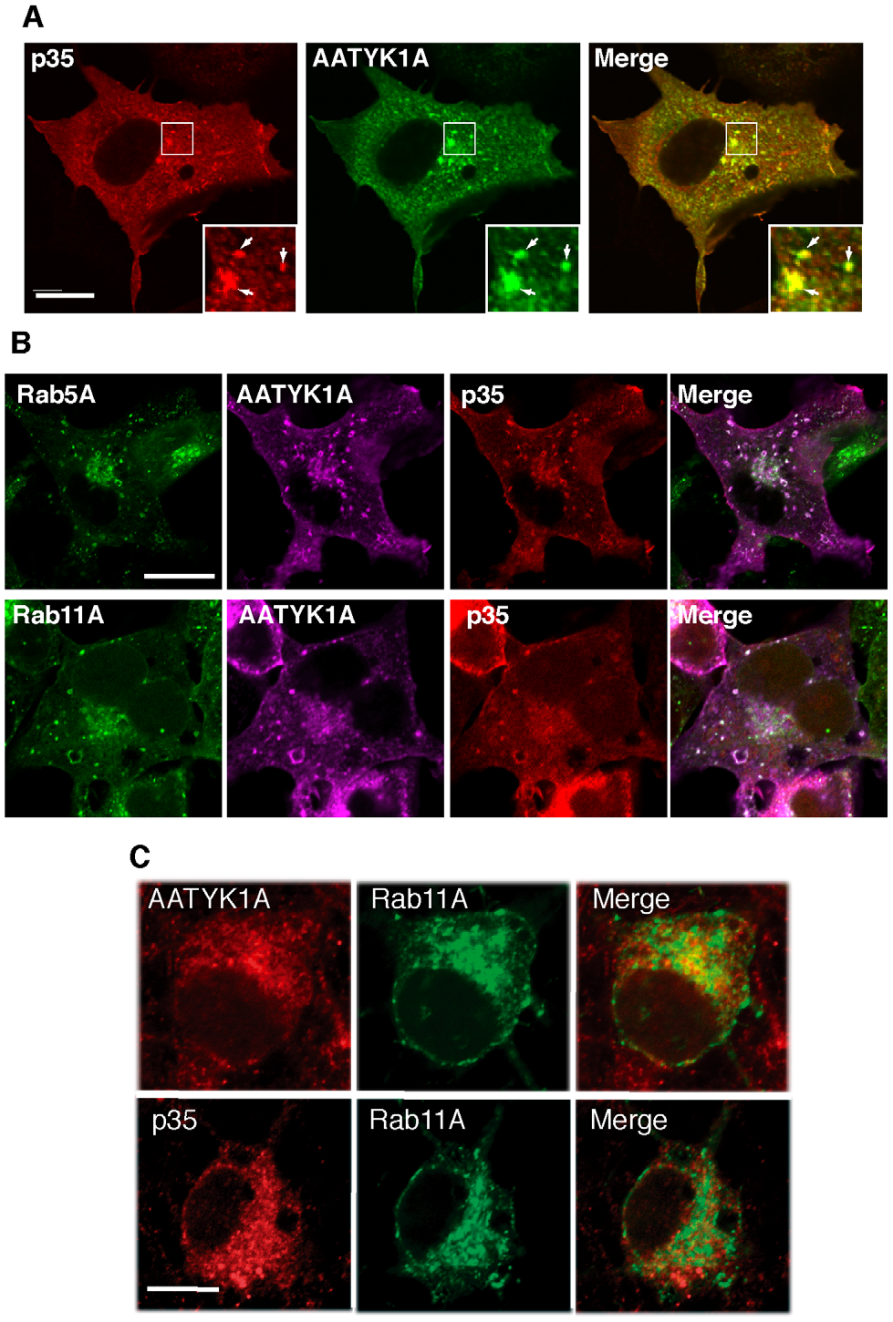
Colocalization of p35 with AATYK1A in early and recycling endosomes. (A) Colocalization of AATYK1A and p35 in COS-7 cells. COS-7 cells were transfected with AATYK1A-Myc together with p35 and Cdk5. AATYK1A and p35 were detected by immunostaining with the anti-Myc antibody and anti-p35 antibody, followed by incubation with Alexa 488-conjugated anti-mouse IgG and Alexa 548-conjugated anti-rabbit antibody, respectively. A merged image is shown in the right panel. Insets represent higher magnifications and arrows indicate the colocalization. Scale bar, 20 µm. (B) Localization of AATYK1A and p35 in early and recycling endosomes. COS-7 cells were transfected with AATYK1A-Myc, p35, Cdk5, and either EGFP-Rab5A (as an early-endosome marker) or EGFP–Rab11A (as a recycling-endosome marker). After 24 h of transfection, cells were fixed and stained with anti-Myc and anti-p35 antibodies, as described above, and were observed using a confocal microscope. Scale bar, 10 µm. (C) Localization of AATYK1 and p35 in endosomes in cultured cortical neurons. Rat brain cortical neurons at DIV5 were transfected with EGFP-Rab11A (middle panels). The cells were immunostained with anti-AATYK1 and p35 (C19) 24 h after transfection, followed by Alexa 546-conjugated anti-rabbit secondary antibody (left panels). Merge is shown in right panels. Bar, 10 µm.

We compared the cellular distribution of AATYK1A with that of p35 in COS-7 cells coexpressing both proteins, as their differential localization has been reported, i.e., Cdk5/p35 at the Golgi apparatus and plasma membrane [17], [18], [19] and AATYK1A mainly at recycling endosomes [16]. The coexpression of AATYK1A and p35 in COS-7 cells led to a punctate staining for p35 in the perinuclear region and cell periphery (Fig. 2A, left panel), as reported previously [17], [18], [19]. AATYK1A also exhibited localization in perinuclear regions (Fig. 2A, middle panel). Higher magnification of the perinuclear region is shown in insets. The merged image depicts their colocalization clearly (arrows in insets of Fig. 1A). To determine whether these proteins were both present in endosomes, AATYK1A and p35 were coexpressed with the endosome markers EGFP-Rab5A (for early endosomes) and EGFP-Rab11A (for recycling endosomes) (Fig. 2B). AATYK1A and p35 both colocalized with early and recycling endosomes, which were labeled with Rab5A and Rab11A, respectively. These data indicate that AATYK1A associates with p35 in early and recycling endosomes in COS-7 cells.

Localization of AATYK1A and p35 in recycling endosomes was next examined in neurons. At first, we tested the localization of exogenously expressed p35 in recycling endosomes using Alexa 546-transferrin (Tf), which is transported to recycling endosomes when incorporated into cells. At 2 h after treatment, Tf accumulated at the perinuclear region, where p35 was strongly labeled (data not shown). To further confirm the localization of endogenous AATYK1A and p35 in recycling endosomes, we compared the staining with anti-AATYK1 or anti-p35 antibodies with EGFP-Rab11A transfected. Rab11A was detected at the perinuclear regions (Fig. 2C) as was reported previously [20]. Both AATYK1A and p35 showed stronger staining at the perinuclear region, and some of them were overlapped with Rab11A (Fig. 2C), indicating the localization of AATYK1 and p35 in recycling endosomes in neurons.

### Phosphorylation of AATYK1A at Ser34 by Cdk5

**Figure 3:**
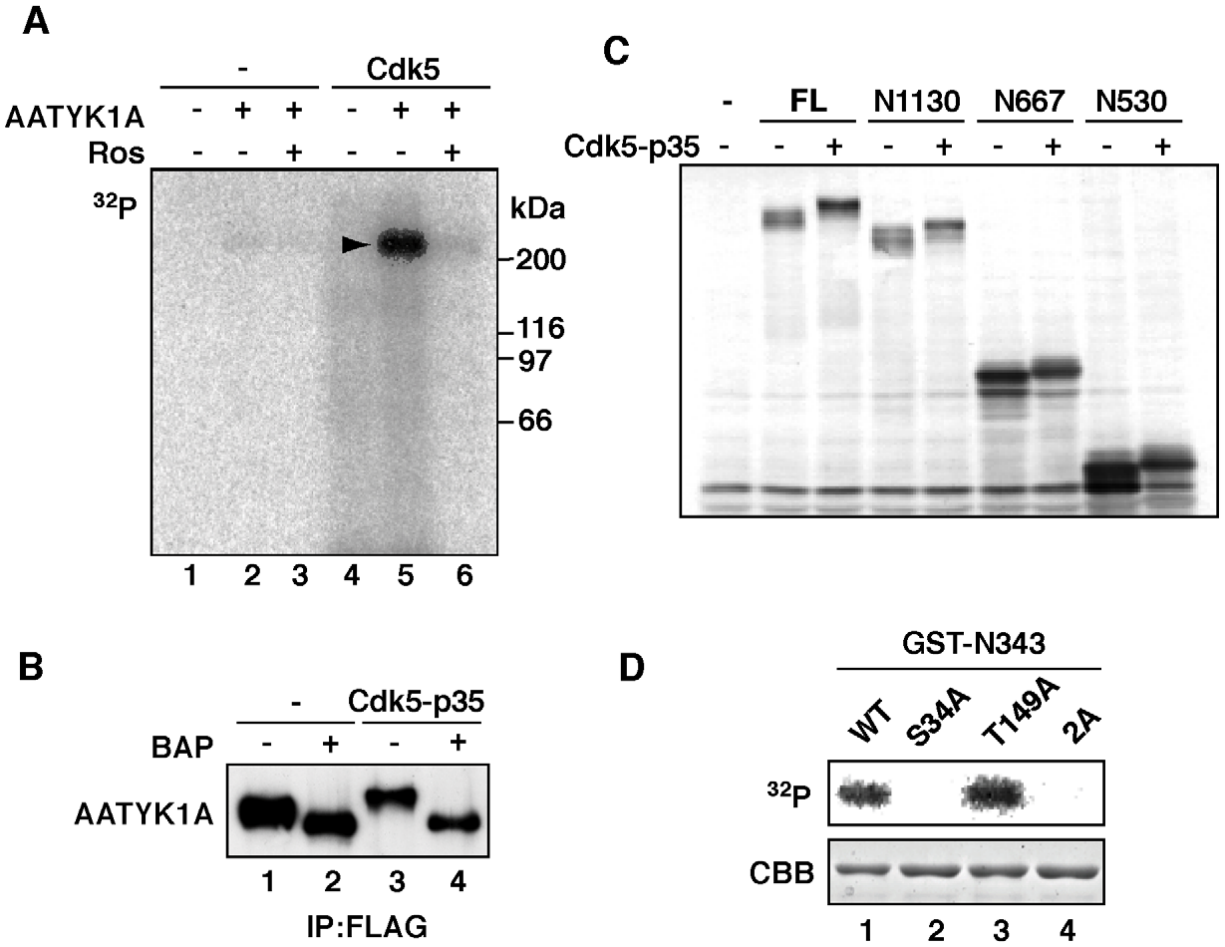
Phosphorylation of AATYK1A at Ser34 by Cdk5. (A) *In vitro* phosphorylation of AATYK1A by Cdk5/p35. AATYK1A-Flag was expressed in HEK293 cells and isolated by immunoprecipitation using the anti-Flag antibody. AATYK1A-Flag was incubated with Cdk5/p35 and [γ-^32^P]ATP in the presence or absence of 50 µM roscovitine (Ros) at 35°C for 30 min. The arrowhead indicates the phosphorylation of AATYK1A. (B) Dephosphorylation of AATYK1A by treatment with alkaline phosphatase. AATYK1A-Flag expressed in the presence (+) or absence (–) of Cdk5/p35 in HEK293 cells was isolated by immunoprecipitation using the anti-Flag antibody. Immunoprecipitates were treated with bacterial alkaline phosphatase (BAP) at 37°C for 30 min and immunoblotted with the anti-Flag antibody. (C) Phosphorylation of amino-terminal fragments of AATYK1A by Cdk5. The full-length (FL) and amino-terminal fragments of AATYK1A-Myc, N1130, N667, and N530, were expressed in HEK293 cells in the presence (+) or absence (–) of Cdk5/p35. HEK293 cell extracts were immunoblotted using the anti-Myc antibody. (D) Ser34 is a Cdk5 phosphorylation site of AATYK1A. The GST-N343 fragment or its Ala mutant at Ser34 (S34A), Thr149 (T149A), or both (2A) was incubated with purified Cdk5/p35 in the presence of [γ-^32^P]ATP at 35°C for 30 min. Phosphorylation was detected using autoradiography after SDS–PAGE. Coomassie Brilliant Blue (CBB) staining of GST-N343 is shown in the lower panel.

As shown in lane 5 of Figure 1A, AATYK1A exhibited a slower mobility on SDS–polyacrylamide gel electrophoresis (SDS–PAGE) when coexpressed with Cdk5/p35 in HEK293 cells. This result suggests that full-length AATYK1A was phosphorylated by Cdk5/p35. To confirm this hypothesis, we incubated AATYK1A with purified Cdk5/p35 *in vitro* in the presence of [γ-^32^P]ATP. AATYK1A was labeled strongly with ^32^P after incubation with Cdk5/p35 (Fig. 3A, lane 5) and this labeling was inhibited by roscovitine, which is a Cdk5 inhibitor (Fig. 3A, lane 6). Cellular phosphorylation was also examined in HEK293 cells cotransfected with AATYK1A and Cdk5/p35. The upward shift of AATYK1A induced by cotransfection with Cdk5/p35 was reversed by alkaline-phosphatase treatment (Fig. 3B, lanes 3 and 4), which suggests that the upward shift of AATYK1A is due to Cdk5/p35-mediated phosphorylation.

To identify the Cdk5/p35 phosphorylation site of AATYK1A, we coexpressed several AATYK1A carboxy-terminal truncation mutants in HEK293 cells with Cdk5/p35 and examined their phosphorylation by band shift (Fig. 3C). All truncation mutants, i.e., AATYK1A amino-acid residues 1–1130 (N1130), 1–667 (N667), and 1–530 (N530), were shifted upward after cotransfection with Cdk5/p35, which suggests the presence of a Cdk5 phosphorylation site in the amino-terminal region of AATYK1A. There are five (Ser/Thr)Pro Cdk5 consensus phosphorylation sequences in N530. We were interested in Ser34, which is the only amino-terminal SP sequence upstream of the kinase domain, because the region includes several functional amino acids, which include tyrosine phosphorylation sites and palmitoylation sites [16]. We constructed an amino-terminal fragment comprising 343 amino acids of AATYK1A (GST-N343) in which the (S/T)P sequences were reduced to two. We incubated GST-N343 with Cdk5/p35 in the presence of [γ-^32^P]ATP (Fig. 3D). GST-N343 was phosphorylated. In contrast, its Ala mutant at Ser34 (S34A) was not phosphorylated (Fig. 3D, lane 2). The Ala mutant at Thr149 was phosphorylated by Cdk5/p35, similarly to the unmutated fragment (Fig. 3D, lane 3), which indicates that Ser34 is a Cdk5 phosphorylation site in N343.

### *In vivo* phosphorylation at Ser34 of AATYK1

**Figure 4:**
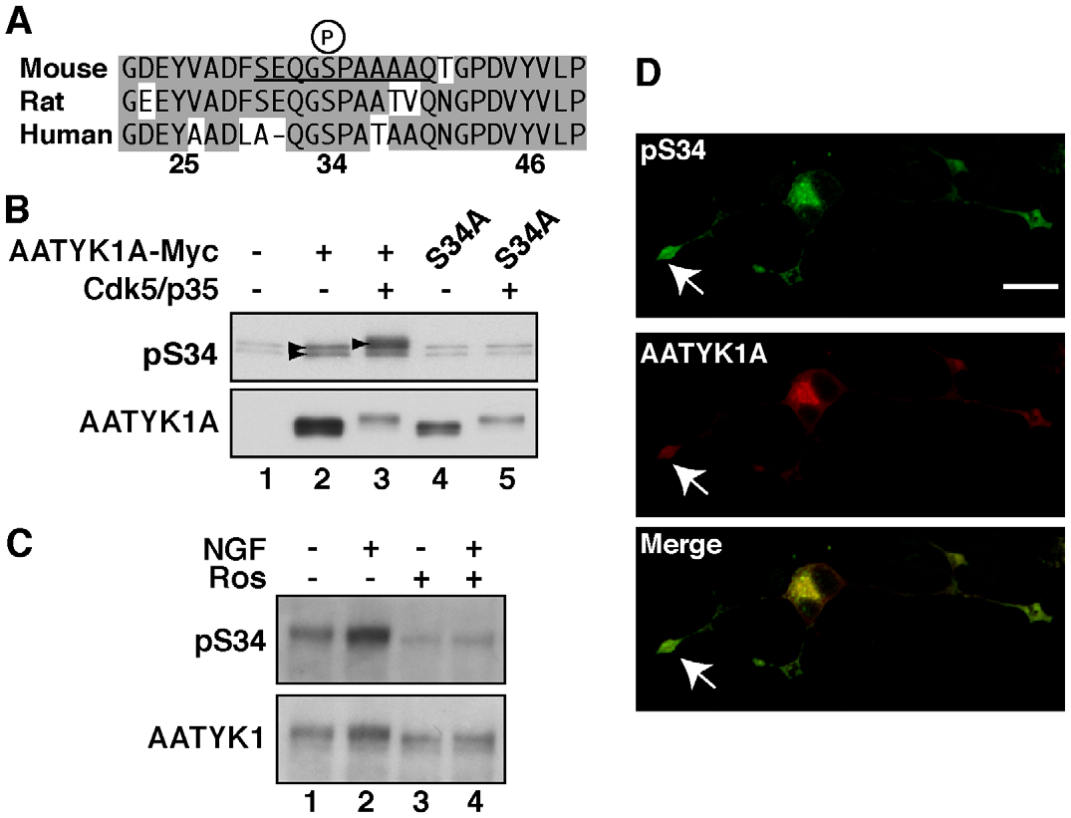
Generation of the anti-pSer34-specific antibody and Ser34 phosphorylation of AATYK1 in PC12D cells. (A) Amino-acid sequences of mouse, rat, and human AATYK1A around Ser34. A synthetic peptide corresponding to the mouse AATYK1A amino-acid residues 29–39 (the Ser34 phosphorylation site is underlined) was used for rabbit immunization. (B) Specificity of the anti-pS34 antibody. COS-7 cells were transfected with AATYK1A or its S34A mutant in the presence (+) or absence (–) of Cdk5/p35. Cell extracts were immunoblotted with the anti-pS34 antibody or anti-Myc antibody for AATYK1A. (C) Phosphorylation of AATYK1 at Ser34 in PC12D cells. PC12D cells were treated with 50 ng/ml NGF for 24 h in the presence or absence of 20 µM roscovitine (Ros). AATYK1 was immunoprecipitated in PC12D cells using the anti-AATYK1 antibody and was subjected to immunoblotting with the anti-pS34 or anti-AATYK1 antibodies. (D) Immunofluorescent staining of PC12D cells using the anti-pS34 antibody. PC12D cells expressing AATYK1A-Myc were treated with NGF for 24 h and double labeled with the anti-pS34 (top panel) and anti-Myc (AATYK1A, middle panel) antibodies. A merged image is shown in the lower panel. The growth cone is indicated by an arrow. Scale bar, 20 µm.

To investigate the *in vivo* phosphorylation of Ser34 of AATYK1A, we raised a phospho-specific AATYK1A antibody using a synthetic phosphopeptide corresponding to mouse AATYK1A residues around Ser34 (Fig. 4A, underlined). The site was conserved among the mammalian AATYK1A of mouse, rat, and humans, although the amino-terminal amino acids of human AATYK1A were somewhat different from those of mouse and rat AATYK1A. The anti-pSer34 antibody reacted with AATYK1A (Fig. 4B, arrowheads in lanes 2 and 3) but not with the S34A mutant in COS-7 cells (Fig. 4B, lanes 4 and 5). The fact that AATYK1A S34A was also shifted upward after cotransfection with Cdk5/p35 implies that AATYK1A should have other Cdk5 phosphorylation site(s), in addition to Ser34 (Fig. 4B, lane 5). The faint bands around AATYK1A S34A were nonspecific reactions, which were also found in untransfected COS-7 cells (Fig. 4B, lane 1). The reaction to AATYK1A was remarkably enhanced by coexpression with Cdk5/p35 (Fig. 4B, lane 3). Next, we addressed Ser34 phosphorylation in neuronal cells. For this, we chose PC12D cells, which express both Cdk5/p35 and AATYK1 [1], [21]. PC12D cells, a subclone of PC12 cells, extend neurites more promptly in response to nerve growth factor (NGF) [22], [23]. We use the term AATYK1 henceforth for endogenous AATYK1 in PC12D cells and brains, as AATYK1A is not distinguishable from AATYK1B using immunoblotting or immunofluorescent staining. AATYK1 and its Ser34 phosphorylation were detected in PC12D cells before NGF application (Fig. 4C, lane 1); however, NGF treatment increased the expression and phosphorylation of AATYK1 significantly (Fig. 4C, lane 2). Roscovitine suppressed the phosphorylation of Ser34 and the electrophoretic mobility shift of AATYK1 (Fig. 4C, lanes 3 and 4). The localization of AATYK1A phosphorylated at Ser34 was examined using immunostaining of transfected PC12D cells, in which we could obtain specific staining with anti-pSer34 antibody. Phospho-AATYK1A was strongly detected at the growth cones of extended neurites and in the cell body of NGF-treated cells (arrow in Fig. 4D). These results indicate that Cdk5 phosphorylates AATYK1 at Ser34 at the tip of neurites in PC12D cells.

**Figure 5:**
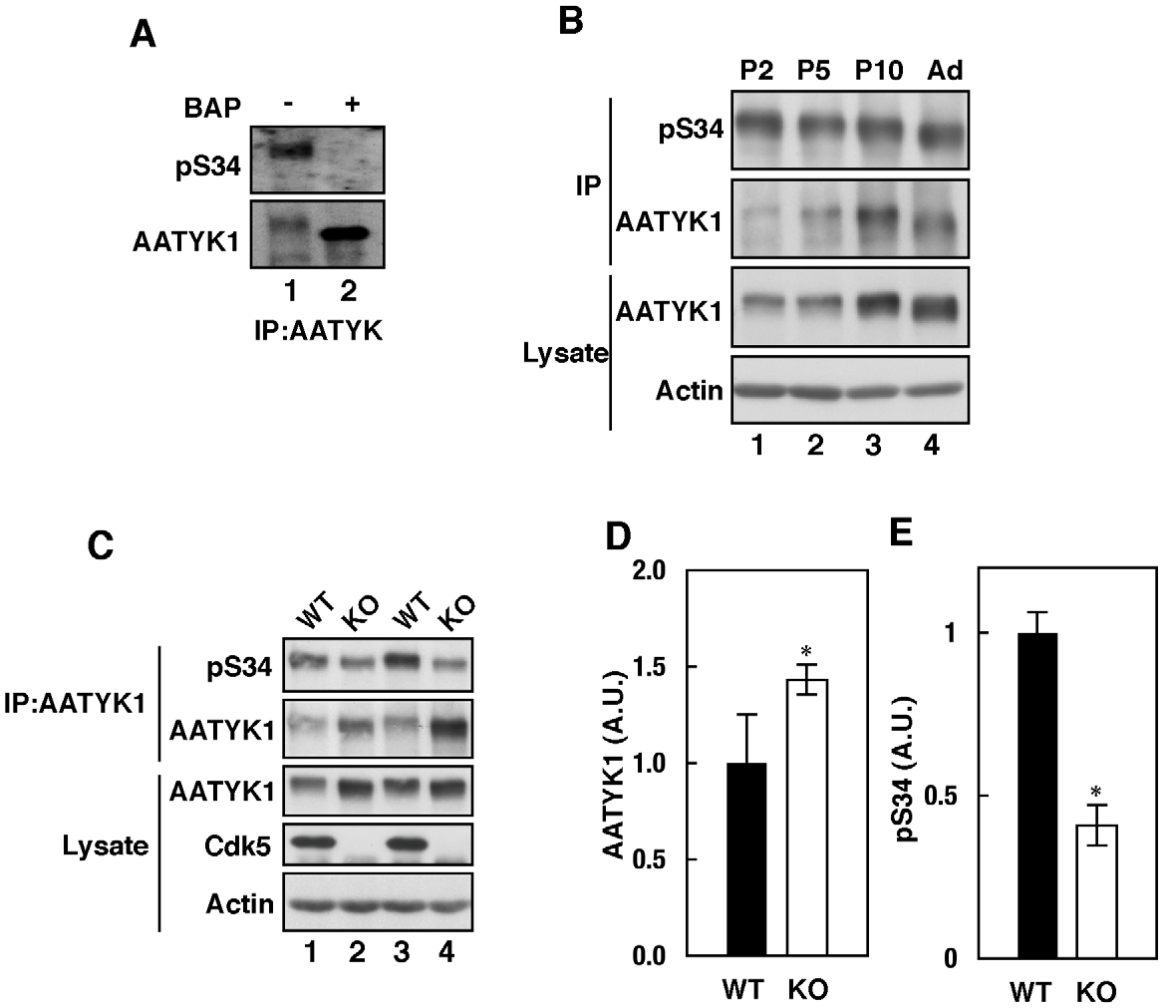
Cdk5-mediated *in vivo* Ser34 phosphorylation. (A) Phosphorylation of AATYK1 in mouse brain. AATYK1 was immunoprecipitated from mouse brain extracts and incubated in the presence (+) or absence (–) of bacterial alkaline phosphatase (BAP). The anti-pS34 reaction is shown in the upper panel and the anti-AATYK1 reaction is shown in the lower panel. (B) Phosphorylation of AATYK1 in mouse brain during early postnatal development. AATYK1 was immunoprecipitated from mouse brain extracts at P2, P5, P10, and six weeks of age (Ad) using the anti-AATYK1 antibody. The immunoprecipitates were immunoblotted using the anti-pS34 (top panel) and anti-AATYK1 (second panel) antibodies. Immunoblots of brain extracts using anti-AATYK1 and anti-actin antibodies are also shown in the lower panels. (C) Phosphorylation of AATYK1 at Ser34 in Cdk5^–/–^ mouse brain. AATYK1 was immunoprecipitated from brain extracts of Cdk5^–/–^ mice at embryonic day 18.5 (E18.5) and immunoblotted using the anti-pS34 antibody (top panel). Immunoblots of the brain extracts using anti-AATYK1 (third panel), anti-Cdk5 (fourth panel), and anti-actin (bottom panel) antibodies. Quantification of AATYK1 and pS34 is shown in (D) and (E), respectively. Bars indicate the means ± S.E. of three independent experiments (n = 3; * *P*<0.05; *t* test).

The anti-pS34 antibody recognized AATYK1 immunoprecipitated from a mouse brain extract at a molecular weight ∼200 KDa (Fig. 5A, lane 1). Dephosphorylation with alkaline phosphatase abolished this reaction (Fig. 5A, lane 2), indicating that AATYK1 is phosphorylated at Ser34 in mouse brain. Expression of AATYK1 increased gradually from P2 to adult age (10 weeks). Ser34 phosphorylation was also detected in brains at P2 and decreased by P10, when normalized to the expression levels of AATYK1 (Fig. 5B).

To confirm that Cdk5 phosphorylated AATYK1 at Ser34 *in vivo*, we examined the phosphorylation of AATYK1 in Cdk5-deficient mouse brains. Interestingly, expression of AATYK1 was increased by 1.43-fold in Cdk5-deficient brain compared with wild-type mouse brain (Fig. 5C, lanes 2 and 4; and Fig. 5D). In Cdk5-deficient brain, AATYK1 exhibited increased electrophoretic mobility (Fig. 5C) and decreased immunoreactivity to the anti-pS34 antibody (Fig. 5C and E), which suggests that Cdk5 phosphorylates Ser34 of AATYK1 in the mouse brain.

### Ser34 phosphorylation inhibited the tyrosine phosphorylation of AATYK1A

**Figure 6:**
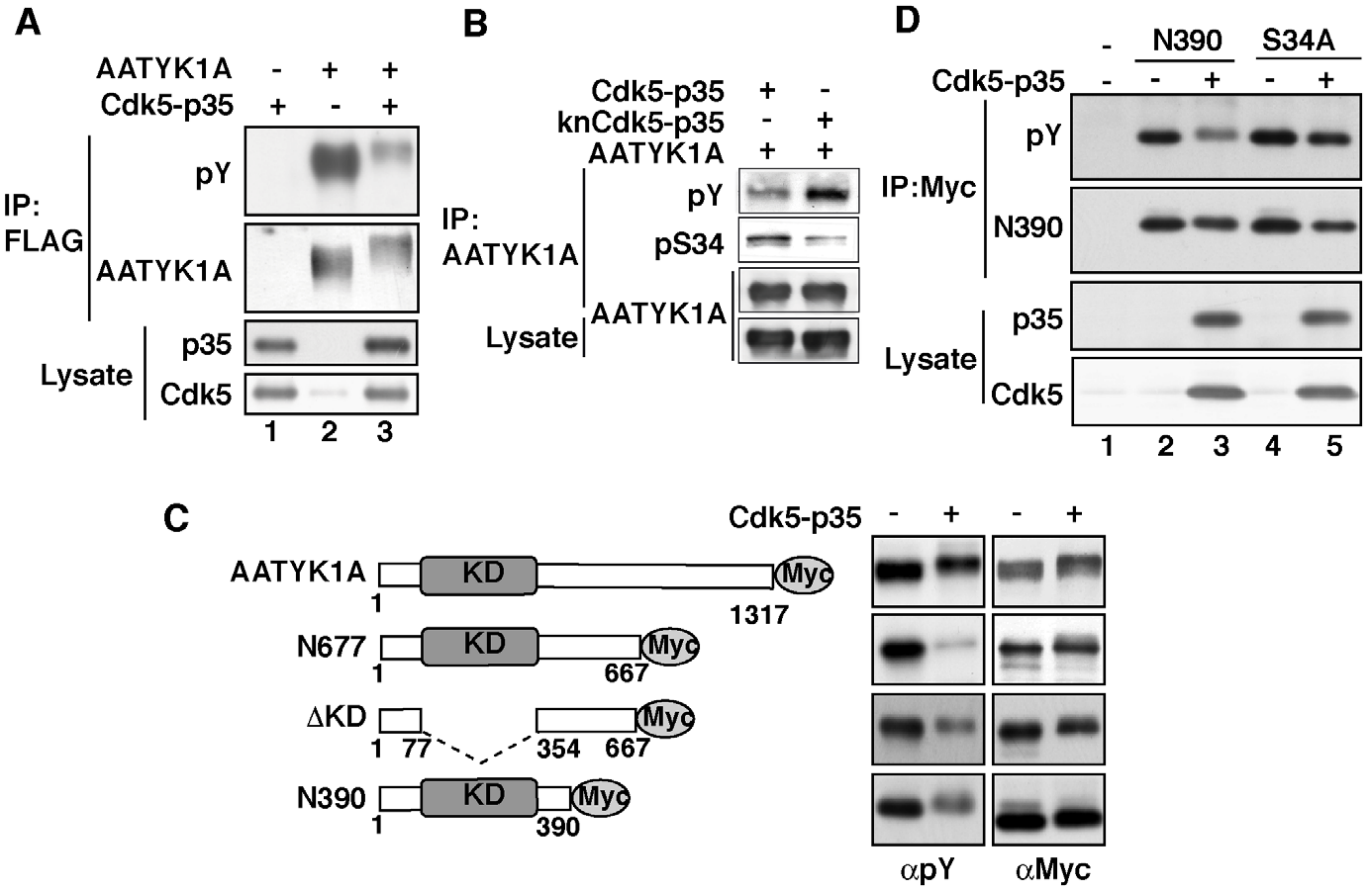
Phosphorylation of AATYK1A by Cdk5 suppressed its tyrosine phosphorylation. (A) Effect of Cdk5/p35 on the tyrosine phosphorylation of AATYK1A. AATYK1A-Flag was transfected into HEK293 cells, alone or with Cdk5/p35. Twenty-four hours after transfection, cells were treated with 50 µM pervanadate for 20 min. AATYK1A was immunoprecipitated with the anti-Flag antibody and examined for tyrosine phosphorylation using immunoblotting with the anti-phospho-Tyr antibody (pY). (B) COS-7 cells expressing AATYK1A and Cdk5/p35 or knCdk5/p35 were treatment with 100 µM pervanadate for 5 min. After immunoprecipitation with the anti-AATYK1A antibody, their tyrosine phosphorylation was examined using the anti-phospho-Tyr antibody (pY) and Ser34 phosphorylation was detected in the immunoprecipitates with anti-pS34 antibody. (C) Tyrosine phosphorylation of AATYK1A and of its deletion mutants, N677, ΔKD, or N390. AATYK1A or its deletion mutants was transfected into COS-7 cells, alone (–) or with Cdk5/p35 (+). After treatment with 100 µM pervanadate for 5 min, the tyrosine phosphorylation of AATYK1A was examined after immunoprecipitation with the anti-Myc antibody. (D) N390 or N390-S34A (S34A) was expressed in COS-7 cells, alone (–) or with Cdk5/p35 (+). Tyrosine phosphorylation was induced by the pervanadate treatment at 100 µM for 5 min. After immunoprecipitation with the anti-Myc antibody, their tyrosine phosphorylation was examined using the anti-phospho-Tyr antibody (pY).

We reported previously on the Fyn- or Src-mediated tyrosine phosphorylation of AATYK1A [16]. We hypothesized that Cdk5-mediated phosphorylation of AATYK1A inhibits its tyrosine phosphorylation. The coexpression of AATYK1A with Cdk5/p35 in COS-7 cells led to a significant decrease in the pervanadate-induced tyrosine phosphorylation of AATYK1A (Fig. 6A, lane 3). The effect of Cdk5 activity on tyrosine phosphorylation is clearly shown in Fig. 6B, in which wild type or kinase negative (kn) Cdk5 was coexpressed with AATYK1A. Coexpression of knCdk5 increased tyrosine phosphorylation together with decreased Ser34 phosphorylation. To identify the tyrosine phosphorylation sites, we analyzed the tyrosine phosphorylation of several deletion mutants of AATYK1A, N667, ΔKD, and N390, which were coexpressed with Cdk5/p35 (Fig. 6C, left panel). Inhibition of tyrosine phosphorylation was observed for all deletion mutants of AATYK1A (Fig. 6C, right panel), which suggests the presence of tyrosine phosphorylation site(s) in N390. To confirm that Ser34 phosphorylation inhibited the tyrosine phosphorylation of AATYK1A, N390 or its Ala mutant at Ser34 (N390-S34A) was expressed together with Cdk5/p35 in COS-7 cells and their tyrosine phosphorylation was examined after pervanadate treatment (Fig. 6D). Inhibition of tyrosine phosphorylation was observed exclusively for N390 coexpressed with Cdk5/p35 (and not for N390-S34A). These results indicate that the Cdk5-mediated Ser34 phosphorylation suppresses the tyrosine phosphorylation of AATYK1A at tyrosine residues located in N390.

## Discussion

We investigated the interaction of AATYK1A, a major isoform of AATYK1 that is expressed in brain, with Cdk5/p35. We observed the *in vivo* binding of AATYK1A to Cdk5/p35 using anti-AATYK1 coimmunoprecipitation from HEK293 cell extracts and mouse brain extracts. In COS-7 cells, these proteins colocalized in early and recycling endosomes labeled with Rab5A and Rab11A, respectively. Cdk5/p35 phosphorylated AATYK1A at Ser34 *in vitro* and *in vivo*. This Cdk5-mediated phosphorylation decreased the tyrosine phosphorylation of AATYK1A. Considering that AATYK2/cprk, a member of the AATYK family that exhibits a Cdk5-phosphorylation-mediated decrease in tyrosine phosphorylation, is involved in endosomal trafficking, the Ser34 and/or tyrosine phosphorylation of AATYK1A may also play a role in regulating endosomal-membrane and -protein transport.

The observation of cellular colocalization and physical binding are required to demonstrate a cellular interaction between two components. AATYK1A binds to membranes via palmitoylation at its three amino-terminal cysteine residues [16] and Cdk5/p35 associates with membranes via myristoylation of p35 [18], [19]. Although both proteins localize in the perinuclear region, the precise organelles where they reside are different; AATYK1A is located in recycling endosomes [16] and Cdk5/p35 is located in the Golgi apparatus [17], [18]. We examined the cellular localization of AATYK1A previously using various endosomal and Golgi markers and showed its presence mainly in Rab11A-positive recycling endosomes (but not in the Golgi) [16]. In contrast, the localization of Cdk5 in endosomes has not been examined, although its association with Golgi and plasma membranes has been demonstrated. Here, the coexpression of these two proteins in COS-7 cells allowed us to confirm that they colocalized in early and recycling endosomes. A fraction of Cdk5/p35, which is distributed widely in various membranous compartments, may associate with AATYK1A in early-to-recycling endosomes.

Cdk5/p35 phosphorylated AATYK1A *in vitro* and *in vivo*. The phosphorylation of AATYK1 was increased in PC12D cells after NGF treatment, which stimulates Cdk5 activity, and was elevated in mouse brains during early postnatal days, when Cdk5 activity is high. AATYK1A is a large protein that comprises 1,317 amino acids and contains 36 (S/T)P Cdk5 phosphorylation consensus sequences, most of which are located in the carboxy-terminal tail domain. We observed that the middle and tail regions of AATYK1A were phosphorylated by Cdk5/p35 (data not shown), which suggests that some of these carboxy-terminal (S/T)P sites may be phosphorylated by Cdk5/p35. The finding that AATYK1A S34A was shifted upward after coexpression with Cdk5/p35 in COS-7 cells is consistent with this idea. However, we focused on the amino-terminal phosphorylation of AATYK1A because the amino-terminal region upstream from the kinase domain seems to be one of the important functional domains of AATYK1A, as it contains the palmitoylation sites Cys 4, 6, and 7 and the tyrosine phosphorylation sites Tyr25 and Tyr46 [16]. The Cdk5-phosphorylation site (Ser34) lies between the two Tyr phosphorylation sites. Notably, Cdk5-mediated Ser34 phosphorylation suppressed the tyrosine phosphorylation of AATYK1A. Although we did not determine whether phosphorylation at these Tyr residues is affected by Cdk5 phosphorylation at Ser34, this seems likely, as suppression of tyrosine phosphorylation occurred in the amino-terminal fragment N390. Introduction of a negative charge into Ser34 may affect subsequent phosphorylation at Tyr residues, which are close to Ser34. These results suggest that Cdk5 and Src family kinases (SFKs) may interplay on AATYK1A in endosomes.

It may be worth pointing out some similarities between AATYK1A and AATYK2, which is also known as LMTK2/KPI-2/BREK/cprk [4], [5], [6], regarding several of the properties described here. AATYK2/cprk was isolated as a p35-binding protein using a yeast two-hybrid system [5], as was the case for AATYK1 [15]. The p35-binding region of both AATYK1A and AATYK2/cprk is adjacent to the kinase domain in the carboxy terminal, which exhibits some amino-acid sequence similarity [1]. AATYK2/cprk is phosphorylated by Cdk5/p35 [5]. This phosphorylation by Cdk5/p35 also reduces the tyrosine phosphorylation of AATYK2/cprk [5]. This result was interpreted based on the decreased kinase activity of AATYK2/cprk, as AATYK2/cprk was suspected to be a tyrosine kinase at the time. However, AATYK2/cprk is now known as a Ser/Thr kinase [4], [6], [24]. Although it remains unknown whether the amino-terminal end of the kinase domain of AATYK2/cprk is phosphorylated, as is that of AATYK1A, there are several possible (S/T)P Cdk5 phosphorylation sites in the region corresponding to Ser34 of AATYK1A. The reduced tyrosine phosphorylation of Cdk5-phosphorylated AATYK2/cprk could be also due to phosphorylation by other Tyr kinases, such as SFKs.

Ablation of AATYK2/cprk using an siRNA approach disrupts the transport of endocytosed membranes from early to recycling endosomes [25], [26], which suggests a function for AATYK2/cprk in endosomal trafficking. In fact, we have recently observed that AATYK1A plays a role in accumulation of recycling endosomes in pericentrosomal compartment in CHO-K1 cells and phosphorylation at Ser34 inhibits its activity (Takano et al., unpublished observation). Interestingly, AATYK1A accumulated in the growth cones of neurites in PC12D cells. Recycling endosomes contribute the membranes and protein trafficking required for neurite outgrowth [27], [28]. On the other hand, Cdk5 suppresses exocytosis of neurotransmitters by preventing Ca^2+^ entry via P/Q type Ca^2+^ channels and suppresses endocytosis of released neurotransmitters via the phosphorylation of dynamin 1 and amphiphysin 1 at the presynaptic terminus [29], [30], [31]. Intracellular vesicle transport uses molecular machinery that is similar to that used in endocytosis and exocytosis. Taking into consideration the association of Cdk5/p35 with cytoplasmic organelles, including early and recycling endosomes, Cdk5/p35 may also participate in vesicle transport. Cdk5/p35 activity is required for neurite outgrowth in cultured neurons and in PC12 cells [24], [32]. Cdk5/p35 and/or SFKs may regulate AATYK1A function in endosomal trafficking via phosphorylation at its Ser34 residue.

## Materials and Methods

### Ethics Statement

Mice were handled in accordance with Riken BSI guidelines and housed in a pathogen-free environment on a 12 h light/dark cycle.

### Chemicals, antibodies, and plasmids

Leupeptin was obtained from the Peptide Institute (Osaka, Japan), 4-(2-aminoethyl)-benzenesulfonyl fluoride hydrochloride (AEBSF or Pefabloc) and Roscovitine were purchased from Wako (Osaka, Japan), and Dulbecco's modified Eagle's medium (D-MEM) and nerve growth factor (NGF) were from Sigma (St. Louis, MO). The PolyFect transfection reagent was obtained from Qiagen (Hilden, Germany) and Lipofectamine 2000 was from Invitrogen (Carlsbad, CA).

The antibodies used in this study were as follows: anti-Flag (M2) and anti-actin (Sigma); monoclonal (9E10) and polyclonal anti-Myc, anti-p35 (C-19), and anti-Cdk5 (DC17) (Santa Cruz Biotechnology, Santa Cruz, CA); Alexa 488-, 546-, and 647-conjugated secondary antibodies to mouse or rabbit IgG (Invitrogen); and anti-phospho-Tyr (4G10) (Upstate Biotechnology, Lake Placid, NY). The anti-AATYK1 antibody was described previously [16]. The anti-phospho-Ser34 (anti-pS34) antibody for AATYK1 was generated via immunization of rabbits with the pSer34 peptide FSEQGpSPAAAAC conjugated to keyhole limpet hemocyanin. The resulting antibody was affinity purified using a nonphosphorylated peptide-conjugated column negatively and a phosphorylated peptide-conjugated column positively.

The mammalian cell-expression plasmids used in this study were as follows: AATYK1A-Flag and AATYK1A-Myc-His [16], pCMV5-Cdk5 and pCMV5-p35 [33], and EGFP-Rab5A and EGFP-Rab11A [34], [35]. The Ala mutants of AATYK1 at Ser34 or Thr149 were constructed using the QuikChange Site-Directed Mutagenesis kit (Stratagene, La Jolla, CA), according to the manufacturer's protocol. GST-N343, which consisted of AATYK1A amino-acid residues 1–343 (N343), was inserted into pGEX 4T-1 (GE Healthcare) after PCR amplification using the AATYK1A cDNA as a template.

### *In vitro* phosphorylation

Cdk5/p35 was purified from Sf-9 cells [36]. AATYK1A-Flag or GST-N343 was incubated with Cdk5/p35 in kinase buffer (10 mM MOPS, pH 6.8, 2 mM MgCl_2_, 0.1 mM EGTA, and 0.1 mM EDTA) in the presence of 0.1 mM [γ-^32^P]ATP for 30 min at 35°C. Phosphorylation was detected using autoradiography after SDS–polyacrylamide gel electrophoresis (PAGE). Tyrosine phosphorylation of the AATYK1A constructs was assessed in COS-7 cells using immunoblotting with the anti-phospho-Tyr (4G10) antibody after immunoprecipitation.

### Cell culture, transfection, and immunoprecipitation

HEK293 and COS-7 cells were maintained in D-MEM containing 10% fetal bovine serum, 100 U/ml penicillin, and 0.1 mg/ml streptomycin [18]. Transfection into COS-7 cells or HEK293 cells was performed using the PolyFect transfection reagent or Lipofectamine 2000, according to the manufacturer's instructions. PC12D cells, which were provided by Dr. Mamoru Sano from the Kyoto Prefectural University of Medicine, were maintained in D-MEM containing 10% fetal bovine serum, 5% horse serum, 100 U/ml penicillin, and 0.1 mg/ml streptomycin [22], [23]. Transfection into PC12D cells was performed using Lipofectamine 2000. Brains of postnatal day 2 (P2) to six-weeks-old ICR mice (Japan SLC, Shizuoka Japan) were homogenized in HEPES buffer (20 mM HEPES, pH 7.5, 150 mM NaCl, 2 mM MgCl_2_, 1 mM EGTA, 0.4 mM AEBSF, 10 ug/ml leupeptin, and 0.5% Nonidet P-40) and AATYK1 was immunoprecipitated using the anti-AATYK1 antibody. Cerebral cortical neurons were prepared from rat brains at embryonic day 18 as described previously [36]. Cdk5-deficient mice were generated and maintained as described previously [37], [38].

### Immunofluorescent staining and observation

Cells were fixed with 4% paraformaldehyde in phosphate-buffered saline (PBS) for 20 min and permeabilized with 0.1% Triton X-100 in PBS containing 5% normal goat serum. Cells were probed with primary antibodies for 1 h at room temperature or overnight at 4°C. After washing with PBS, cells were stained with Alexa-conjugated secondary antibodies. Fluorescent images were acquired using an LSM5 EXCITER confocal laser-scanning microscope (Carl Zeiss, Oberkochen, Germany).
